# Factors associated with obtaining prescribed safer supply among people accessing harm reduction services: findings from a cross-sectional survey

**DOI:** 10.1186/s12954-024-00928-9

**Published:** 2024-01-06

**Authors:** Heather Palis, Beth Haywood, Jenny McDougall, Chloé G. Xavier, Roshni Desai, Samuel Tobias, Heather Burgess, Max Ferguson, Lisa Liu, Brooke Kinniburgh, Amanda K. Slaunwhite, Alexis Crabtree, Jane A. Buxton

**Affiliations:** 1 Department of Psychiatry, University of British Columbia, 255 Wesbrook Mall, Vancouver, BC V6T 2A1 Canada; 2 British Columbia Centre for Disease Control, 655 W 12th Ave, Vancouver, BC V5Z 4R4 Canada; 3 Coalition of Substance Users of the North (CSUN), Quesnel, Canada; 4 School of Population and Public Health, University of British Columbia, 2206 East Mall, Vancouver, BC V6T 1Z3 Canada; 5 BC Centre on Substance Use, 400-1045 Howe Street, Vancouver, BC V6Z 2A9 Canada; 6 Public Health Agency of Canada, 655 W 12th Ave, Vancouver, BC V5Z 4R4 Canada

**Keywords:** Prescribed safer supply, Safe supply, Safer supply, Harm reduction, Overdose prevention, Drug checking services

## Abstract

**Background:**

With growing rates of unregulated drug toxicity death and concerns regarding COVID-19 transmission among people who use drugs, in March 2020, prescribed safer supply guidance was released in British Columbia. This study describes demographic and substance use characteristics associated with obtaining prescribed safer supply and examines the association between last 6-month harm reduction service access and obtaining prescribed safer supply.

**Methods:**

Data come from the 2021 Harm Reduction Client Survey administered at 17 harm reduction sites across British Columbia. The sample included all who self-reported use of opioids, stimulants, or benzodiazepines in the prior 3 days (*N* = 491), given active use of these drugs was a requirement for eligibility for prescribed safer supply. The dependent variable was obtaining a prescribed safer supply prescription (Yes vs. No). The primary independent variables were access to drug checking services and access to overdose prevention services in the last 6 months (Yes vs. No). Descriptive statistics (Chi-square tests) were used to compare the characteristics of people who did and did not obtain a prescribed safer supply prescription. Multivariable logistic regression models were run to examine the association of drug checking services and overdose prevention services access with obtaining prescribed safer supply.

**Results:**

A small proportion (*n* = 81(16.5%)) of the sample obtained prescribed safer supply. After adjusting for gender, age, and urbanicity, people who reported drug checking services access in the last 6 months had 1.67 (95% CI 1.00–2.79) times the odds of obtaining prescribed safer supply compared to people who had not contacted these services, and people who reported last 6 months of overdose prevention services access had more than twice the odds (OR 2.08 (95% CI 1.20–3.60)) of prescribed safer supply access, compared to people who did not access these services.

**Conclusions:**

Overall, the proportion of respondents who received prescribed safer supply was low, suggesting that this intervention is not reaching all those in need. Harm reduction services may serve as a point of contact for referral to prescribed safer supply. Additional outreach strategies and service models are needed to improve the accessibility of harm reduction services and of prescribed safer supply in British Columbia.

## Background

The COVID-19 pandemic was preceded by an ongoing unregulated drug toxicity emergency in British Columbia (BC), first declared in 2016 [1]. This public health emergency has continued to worsen since 2020 with record unregulated drug toxicity death rates in 2021 (44.2 per 1000,000) and 2022 (42.7 per 100,000) more than doubling the rates in 2019 (19.4 per 100,000) [2].

With growing rates of unregulated drug toxicity death and concerns regarding COVID-19 transmission among people who use drugs, in March 2020, the British Columbia (BC) Ministry of Health and BC Centre on Substance Use issued “Risk Mitigation Guidance” for physicians and nurse practitioners to prescribe opioids, stimulants, and benzodiazepines as alternatives to the unregulated drug supply for people at risk of unregulated drug toxicity events [3, 4]. In July 2021, the Ministry of Health released a prescribed safer supply (PSS) policy directive providing guidance for ongoing prescribing of these medications beyond the Risk Mitigation Guidance, which was introduced as an emergency COVID-19 pandemic-related response [5]. To be consistent with the policy direction in effect at the time of writing, we use the phrase prescribed safer supply (PSS) to refer to pharmaceutical alternatives to the unregulated drug supply that were dispensed under either Risk Mitigation Guidance or PSS policy direction.

Prior to the introduction of Risk Mitigation Guidance and the PSS policy directive, most of the intervention efforts to reduce unregulated drug toxicity events in BC have focused on implementation of harm reduction services. For example, as of February 2023, there are 41 overdose prevention services (OPS) and supervised consumption sites (SCS) in BC, spanning each of the five health regions, 15 of which permit inhalation [6]. Drug checking services (DCS) using fentanyl and benzodiazepine immunoassay strips are available at each of these sites across the province. Some locations offer DCS with Fourier-transform infrared spectroscopy, a technique where a trained technician analyses a submitted sample and can report on abundant components [7]. Results from DCS are always delivered to service users with corresponding and relevant information and advice. While these various harm reduction services help to reduce the risk of harm, the toxicity of the unregulated drug supply means that all people using these drugs face a risk of unregulated drug toxicity events and other drug-related harms. Prior studies have found that harm reduction sites can serve as a point of contact for referral to other health and substance use services and social services such as housing and income assistance [8, 9]. As such, these sites may also serve as a lower barrier point of contact for referral to PSS compared to more traditional medical or substance use treatment settings, thus allowing PSS to reach people who may otherwise not have access to PSS.

To date, understanding of PSS access in BC has been largely determined from province-wide administrative health data. While these data provide estimates of reach by health region, and other demographics such as sex and age, there are other important substance and service use data that are not available from these data sources. For example, patterns of substance use (e.g. substance use type, polysubstance use, frequency of use, route of administration) and sociodemographic information (like gender identity) are poorly captured by administrative data. Furthermore, the extent of reach of PSS among people accessing harm reduction services in BC remains unknown.

This analysis uses data from the 2021 BC Harm Reduction Client Survey (HRCS). The HRCS was first launched in 2012 by the BC Centre for Disease Control as a means of expanding provincial knowledge of harm reduction services access across BC, given the majority of knowledge regarding these services had been derived from urban centres. This study aims to: (1) describe demographic, substance use, and service access characteristics associated with receiving a PSS prescription; and (2) examine the association between last 6-month harm reduction service access and receiving a PSS prescription.

## Methods

### Data sources

**Fig. 1:**
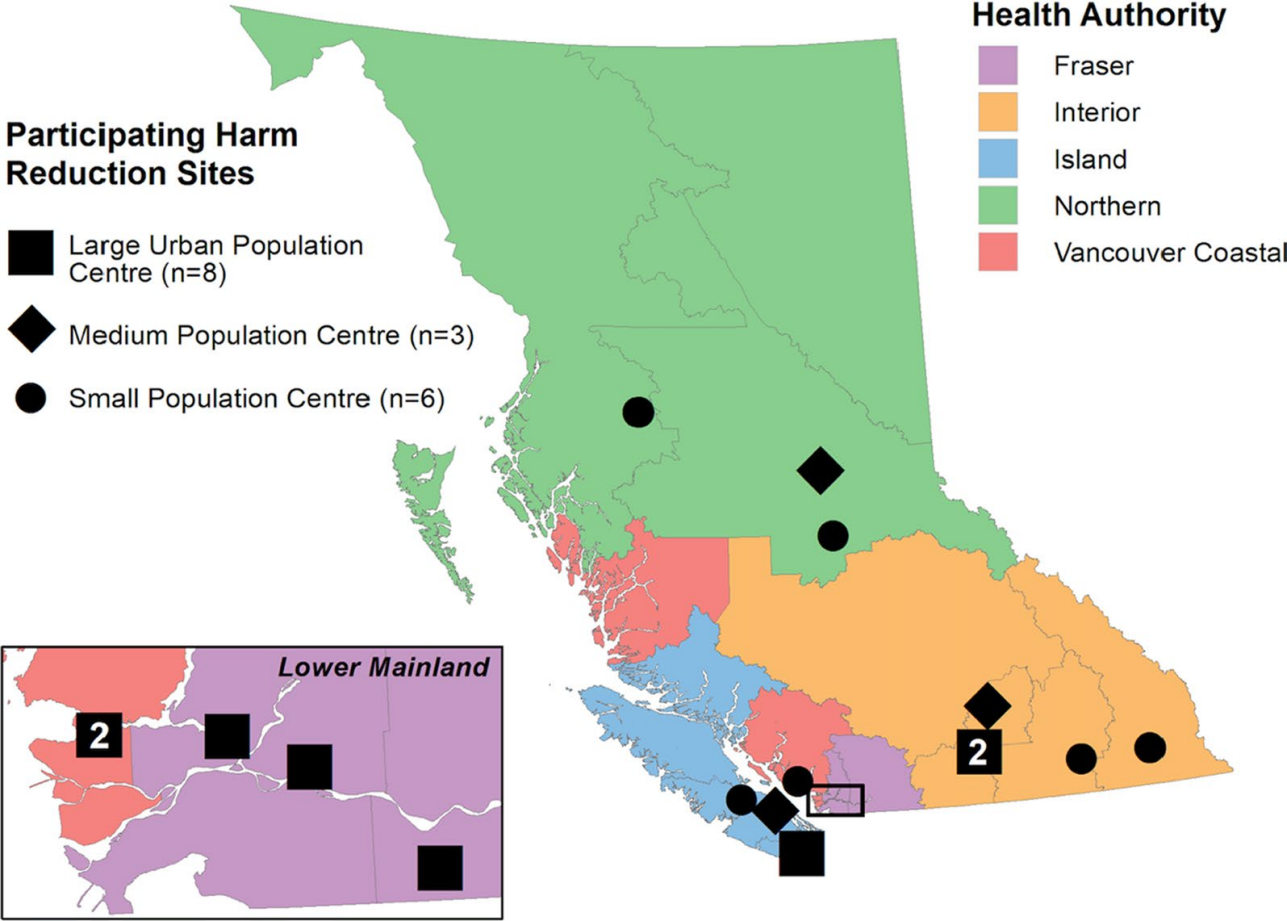
Participating harm reduction sites by health authority region and Statistics Canada Population Centre Classification

Data come from the 2021 HRCS administered at 17 harm reduction sites across BC [10]. Harm reduction sites in the context of this study refer to sites where harm reduction services and supplies are distributed; this includes overdose prevention and drug checking services sites, but also includes shelters and support societies, health clinics, and drug user organizations. Each site operates in a flexible manner, such that participants may attend the sites to access a range of services. For example, while a site may offer an OPS, participants may not necessarily be attending the site to use the OPS (e.g. they could access the site to pick up a naloxone kit). Participating sites cover each health region in BC, including communities in large urban centres (*N* = 8), medium population centres (*N* = 3), and small population centres (*N* = 6) (see Fig. 1). People accessing these sites for any reason who were aged 19 or older who reported the use of illegal substances in the last 6 months were invited to complete this cross-sectional survey. Participants received a $15 honorarium upon completion. All data were collected between March 2021 and January 2022.

### Analytic sample

Given the focus of the analysis on the outcome of receipt of PSS, the analytic sample was defined based on eligibility to receive PSS. In BC, PSS includes prescribed opioids, stimulants, and benzodiazepines (see Table 1 for medications and dosing guidance). These medications were prescribed only to people who were already actively using these drugs. As such, participants were considered eligible for the present analysis if they self-reported the use of illegal opioids, illegal stimulants, or benzodiazepines (legal or illegal, without a prescription) in the past 3 days at the time of survey administration. In the HRCS, questions about current substance use were posed as follows “*Did you use any of these in the last 3 days (Check off all that apply)”* (*N* = 491). This sample represents 91.4% of all 2021 HRCS respondents (*N* = 537).

**Table 1:** Prescribed safer supply drugs and daily dosing guidance

Drug	Instructions
*Opioids*	
M-Eslon (morphine)	Prescribe M-Eslon: 80–240 mg PO BID provided daily (avoid sprinkling doses)
Hydromorphone tablets (Dilaudid)	Prescribe oral hydromorphone: 8 mg tablets (1–3 tabs q1h as needed up to 14 tablets), provided daily
*Stimulants*	
Dextroamphetamine (Dexedrine)	Prescribe Dexedrine: Dexedrine SR (dextroamphetamine) 10–20 mg PO BID provided daily with a maximum dose of 40 mg BID per day AND/OR Dexedrine 10–20 mg IR PO BID-TID with a maximum dose of 80 mg Dexedrine per day
Methylphenidate (Ritalin)	Prescribe methylphenidate: Methylphenidate SR 20–40 mg PO OD with maximum dose of 100 mg/24 h AND/OR Methylphenidate IR 10–20 mg PO BID daily to maximum dose of 100 mg methylphenidate per day
*Benzodiazepines*	
Diazepam (Valium)	Example maintenance dosing: · If the patient describes buying diazepam 10 mg × 3/day then consider starting at 5 mg TID and increasing the dose as needed · If the patient describes using 1–4 “bars” of Xanax, start with clonazepam 0.5–1 mg BID Example maintenance dosing: · If the patient describes buying diazepam 10 mg × 3/day then consider starting at 5 mg TID and increasing the dose as needed
Clonazepam (Klonopin)	Example maintenance dosing: · If the patient describes using 1–4 “bars” of Xanax, start with clonazepam 0.5–1 mg BID

Drugs as listed in BC’s Risk Mitigation Guidance

*BID* twice a day, *TID* three times a day, *PO* by mouth, *OD* once daily, *IR* immediate release, *SR* sustained release, *Q1H* every hour

## Measures

### Dependent variable

The dependent variable was receipt of PSS. Given PSS was a relatively new intervention, a preamble was included in the survey, to ensure the intervention being inquired about was clearly introduced. PSS was described as follows: *“Pandemic prescribing, (sometimes called Risk Mitigation Guidance) allows physicians to prescribe some opioids, stimulants, and benzodiazepines so that people who use drugs can access safer drugs to prevent withdrawal and to allow physical distancing during the COVID-19 pandemic”.*

**Fig. 2:**
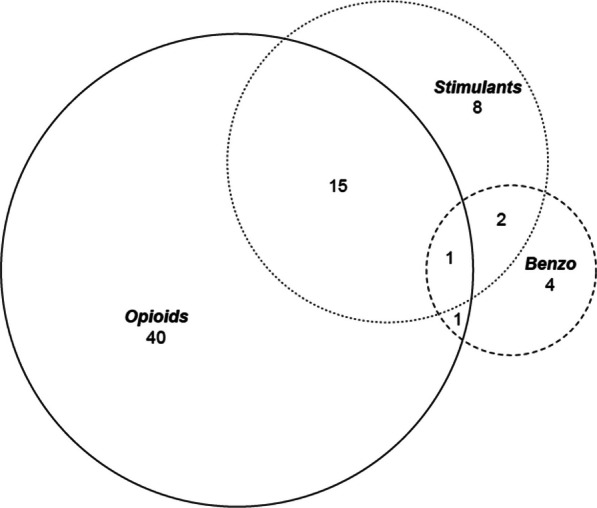
Number of participants receiving one or more prescribed safer supply medication, by medication type. The *N* = 81 PSS recipients include opioids (*N* = 597), stimulants (*N* = 26 = 25), and benzo (*N* = 8). Among the opioid group (*N* = 40 opioid only; *N* = 15 stimulant and opioid; *N* = 31 opioid and benzodiazepine, *N* = 1 benzo, opioid, stimulant). Among the stimulant group (*N* = 8 stimulant only; *N* = 15 stimulant and opioid; *N* = 2 stimulant and benzo, *N* = 1 benzo, opioid, stimulant). Among the benzodiazepine group (*N* = 4 benzodiazepine only; *N* = 2 stimulant and benzo; *N* = 1 opioid and benzodiazepine, *N* = 1 benzo, opioid, stimulant). Of the *N* = 81 who reported receiving PSS, the PSS type was unspecifiednot specified in *N* = 10 cases

Participants were asked about whether they had heard of, or tried to obtain a PSS prescription, and were then asked *“What drugs did you receive a prescription for? (Select all that apply).* Response options were: “Opioids”, “Stimulants”, “Benzodiazepines”, “Other, specify”, or “Prefer not to say”. Using this information, PSS receipt was constructed as a binary variable, with “Yes” being denoted for participants who self-reported receipt of an opioid, stimulant, or benzodiazepine prescription, and “No” being denoted for those who responded no, or prefer not to answer. Participants could self-report receipt of more than one drug. PSS drug types received are reported in Fig. 2.

Data collection in this study overlapped implementation of two separate safer supply policies in the province, with Risk Mitigation Guidance being introduced March 26th, 2020, and the PSS policy directive being introduced July 15th, 2021. As such, when referring to PSS in this analysis, we are referring to receipt of medications as outlined above, which may have occurred in the context of either Risk Mitigation Guidance or prescribed safer supply policy directives.

### Independent variables

The primary independent variables of interest were those reflecting contact with harm reduction services in the last 6 months (i.e. DCS, or OPS). Contact with these services was self-reported, as a binary “Yes” versus “No”.

Independent variables included in the models were: gender (man, woman, transgender and/or gender expansive which included transgender man, transgender women, gender non-confirming, or other not specified); age (categorized according to groupings typically reported in surveillance by BCCDC and the BC Coroners Service (< 30, 30–39, 40–49, ≥ 50); urbanicity (categorized according to Statistics Canada Population Centre Classifications; large urban population centre, medium population centre, and small population centre).

Other variables that were reported to describe the demographic and substance use profiles of HRCS respondents included housing (Stable = living in a private residence, hotel, motel, rooming house, single room occupancy hotel, or social/supportive housing; Unstable = shelter, no regular place to stay (homeless, couch surf, no fixed address); and employment status (Employed = paid full time or part time, or volunteer work; Unemployed = unemployed), health authority of residence (Fraser Health, Interior Health, Island Health, Northern Health, Vancouver Coastal Health), prior 3 days substance use (opioid, stimulant, benzodiazepine), and substance use practices (i.e. last 6-month route of use, frequency of use, using drugs alone, and overdose). These variables are reported in full in Tables 2, 3. Data on Indigenous identity were collected in the HRCS. These data are not presented in this manuscript, as all analyses of PSS access among Indigenous peoples will be led by the First Nations Health Authority and Métis Nation BC.

**Table 2:** Substance use in the last three days by prescribed safer supply medication receipt

	Total *N* = 491 *N* (%)	Did not receive PSS *N* = 410 (83.5%) *N* (%)	Received PSS *N* = 81 (16.5%) *N* (%)	Chi-square *p* value
*Substance Use (Prior 3 day)*				
Any illicit opioid	347 (70.7)	280 (68.3)	67 (82.7)	0.009
Any illicit stimulant	437 (89.0)	366 (89.3)	71 (87.7)	0.671
Crystal meth	385 (78.4)	316 (77.1)	69 (85.2)	0.105
Cocaine	99 (20.2)	93 (22.7)	6 (7.4)	0.002
Crack	140 (28.5)	121 (29.5)	19 (23.5)	0.270
MDMA	30 (6.1)	25 (6.10)	5 (6.2)	0.979
Use any benzodiazepines (yes) (a)	125 (25.5)	98 (23.9)	27 (33.3)	0.075
Xanax	31 (6.3)	25 (6.10)	6 (7.4)	0.658
Other benzos	113 (23.0)	88 (21.5)	25 (30.9)	0.066
Cannabis	232 (47.3)	202 (49.3)	30 (37.0)	0.044
Tobacco	388 (79.0)	319 (77.8)	69 (85.2)	0.136
Alcohol	209 (42.6)	177 (43.2)	32 (39.5)	0.542
*Stimulant and or opioid*				
Neither opioid nor stimulant (benzo only)	3 (0.6)	1 (0.25)	2 (2.5)	0.002
Opioid but not stimulant	51 (10.4)	43 (10.5)	8 (9.9)	
Stimulant but not opioid	141 (28.7)	129 (31.5)	12 (14.8)	
Both	296 (60.3)	237 (57.8)	59 (72.8)	
*Substance use practices*				
*Smoke and/or inject (L6M)*				
Neither	63 (12.8)	56 (13.7)	7 (8.6)	0.001
Inject only	31 (6.3)	27 (6.6)	4 (4.9)	
Smoke only	257 (52.3)	225 (54.9)	32 (39.5)	
Both	140 (28.5)	102 (24.9)	38 (46.9)	
*Frequency of use*				
Every day	342 (69.7)	278 (73.5)	64 (86.5)	0.043
A few times a week	84 (17.1)	75 (19.8)	9 (12.2)	
A few times a month	26 (5.3)	25 (6.61)	1 (1.4)	
*Use drugs alone*				
Never	51 (10.4)	38 (9.64)	13 (16.46)	0.290
Occasionally	157 (32.0)	131 (33.3)	26 (32.9)	
Often	173 (35.2)	145 (36.8)	28 (35.4)	
Always	92 (18.7)	80 (20.3)	12 (15.2)	
Overdose (opioid)	125 (25.5)	98 (26.3)	27 (36.5)	0.076
Overdose (stimulant)	52 (10.6)	45 (12.2)	7 (9.2)	0.461
Overdose (op or stim)	157 (32.0)	126 (30.7)	31 (38.3)	0.184

(a) Benzo use includes intentional use of benzos, and use of non-benzo drugs contaminated with benzodiazepines. Total is based on HRCS respondents who used illicit opioids, illicit stimulants, or any benzodiazepines in the last 3 days (and therefore who would be eligible to receive an opioid, stimulant, or benzodiazepine PSS prescription)

*SRO* single room occupancy, *L6M* last 6 months

**Table 3:** Health service and demographic characteristics by prescribed safer supply medication receipt

	Total *N* = 491 *N* (%)	Did not receive PSS *N* = 410 (83.5%) *N* (%)	Received PSS *N* = 81 (16.5%) *N* (%)	Chi-square *p* value
*Demographics*				
Age				
< 30 years	63 (12.8)	44 (19.7)	19 (23.5)	0.003
30–39 years	128 (26.1)	107 (26.1)	21 (25.9)	
40–49 years	127 (25.9)	102 (24.9)	25 (30.9)	
≥ 50 years	158 (32.2)	144 (35.1)	14 (17.3)	
Unknown	15 (3.1)	13 (3.2)	2 (2.5)	
Gender				
Cis man	307 (62.5)	256 (62.4)	51 (63.0)	0.126
Cis woman	168 (34.2)	144 (35.1)	24 (29.6)	
Transgender and gender diverse	8 (1.6)	5 (1.2)	3 (3.7)	
Missing	8 (1.6)	5 (1.2)	3 (3.7)	
Housing	15 (3.1)			
Stable	267 (54.4)	227 (55.4)	40 (49.4)	0.100
Unstable	210 (42.8)	169 (41.2)	41 (50.6)	
Unknown	14 (2.9)	14 (3.4)	0	
Employment status				
Employed	94 (19.1)	78 (19.0)	16 (19.8)	0.887
Unemployed	367 (74.7)	306 (74.6)	61 (75.3)	
Unknown	30 (6.1)	26 (6.3)	4 (4.94)	
Urbanicity				
Large urban population centre	167 (34.0)	151 (36.8)	16 (19.8)	0.003
Medium population centre	179 (36.5)	149 (36.3)	30 (37.0)	
Small population centre	145 (29.5)	110 (26.8)	35 (43.2)	
*Health authority*				
Fraser health	95 (19.3)	89 (21.7)	6 (7.4)	< 0.001
Interior health	136 (27.7)	121 (29.5)	15 (18.5)	
Island health	110 (22.4)	99 (24.2)	11 (13.6)	
Northern health	85 (17.3)	56 (13.7)	29 (35.8)	
Vancouver coastal health	65 (13.2)	45 (11.0)	20 (24.7)	
L6M service access				
Overdose prevention site	137 (27.9)	104 (28.3)	33 (44.0)	0.007
Drug checking services	192 (39.1)	151 (36.8)	41 (50.6)	0.020
*Opioid agonist treatment*				
Yes	193 (39.3)	140 (34.2)	53 (65.4)	< 0.001
No	234 (47.7)	214 (52.2)	20 (24.7)	
NA, do not use opioids or PNTA	64 (13.0)	56 (13.7)	8 (9.9)	

Total is based on HRCS respondents who used illicit opioids, illicit stimulants, or any benzodiazepines in the last 3 days (and therefore who would be eligible to receive an opioid, stimulant, or benzodiazepine PSS prescription

Stable housing includes the following response options: private residence alone or with someone else, other residence (hotels, motels, rooming houses, single room occupancy (SRO), social/supportive housing, etc.); unstable housing includes shelter, no regular place to stay (homeless, couch surf, no fixed address)

Employed; yes = paid full or part time or volunteer work; No = unemployed. Urbanicity is based on Statistics Canada Population Centre Classification. Participating sites are mapped in Fig. 1

*PNTA* prefer not to answer, *L6M* Last 6 months, *OAT* opioid agonist treatment, *OPS* overdose prevention site

## Data analysis

To address the first objective, descriptive statistics (Chi-square tests) were used to compare the substance use, demographic, health region and substance use services access characteristics of people who did and did not obtain a PSS prescription. Chi-square tests of independence were performed with statistical significance defined as *P* < 0.05. To examine the association between substance use services access and the outcome of PSS receipt, two separate independent variables of interest were explored, OPS access in the last 6 months and DCS access in the last 6 months. These variables were highly correlated.

To investigate the effect of each of these variables on the outcome, independent of one another, separate multivariable models were run for each of these forms of services access. For all models, variables hypothesized to confound the association with PSS receipt were pre-specified (age, sex, and urbanicity). Unadjusted and adjusted multivariable logistic regression models were performed.

## Results

The 2021 HRCS had 537 respondents. Overall, 8.6% (*n* = 46) of participants were excluded from the present analysis, as they did not report the use of opioids, stimulants, or benzodiazepines in the prior 3 days, and thus were not considered eligible to receive PSS. A small proportion (*n* = 81(16.5%)) of HRCS respondents whose survey responses indicated they were eligible for PSS (*n* = 491) received a PSS prescription. Among those receiving a PSS prescription (*n* = 81), 88% (*N* = 71) reported PSS type received. Among those reporting PSS types received, 80.3% reported opioids (*N* = 57), 36.6% reported stimulants (*N* = 26) and 11.3% reported benzodiazepines (*N* = 8). These proportions exceed 100% as some people reported receiving prescriptions for more than one PSS medication. The various combinations of PSS medication types received are presented in Fig. 2.

When considering the substance use profile of respondents, and those who did and did not receive PSS, patterns closely reflected the substances prescribed. For example, most of the PSS prescriptions were for opioids, and it is therefore intuitive that people who received PSS were significantly more likely to report prior 3 days illegal opioid use compared to people who did not (as illegal opioid use was a prerequisite for receipt of an opioid PSS prescription), while overall, there were no differences in reported stimulant use by PSS, when considering stimulant use types, people who received PSS were more likely to report methamphetamine use (85.2% vs. 77.1%, *p* = 0.105) and were significantly less likely to report cocaine use (7.4% vs. 22.7%, *p* = 0.002) compared to people who did not receive PSS.

Overall, approximately 60% of the analytic sample reported the use of both opioids and stimulants in the last 3 days, 10.4% reported opioids but no stimulants, 28.7% reported stimulants but no opioids, and 0.6% reported neither. People who reported receipt of PSS were significantly more likely than those who did not to report the use of both opioids and stimulants (72.8% vs. 57.8%, *p* = 0.002). People who reported PSS receipt were significantly more likely to use by both injection and smoking (46.9% vs. 24.9%, *p* = 0.001) and to report daily illegal substance use (86.5% vs. 73.5%, *p* = 0.043) (Table 2).

People who reported receipt of PSS were significantly younger than those who did not (80.3% vs. 70.7% aged < 50 years, *p* = 0.003) and were significantly more likely to reside in a small population centre (43.2% vs. 26.8%, *p* = 0.003). People who received PSS were significantly more likely to report last 6-month access to an OPS (44.0% vs. 28.3%, *p* = 0.007), and DCS (50.6% vs. 36.8%, *p* = 0.02) (Table 3).

The multivariable logistic regression models demonstrated that OPS and DCS access in the last 6 months was associated with higher odds of PSS receipt. After adjusting for gender, age, and urbanicity, people who had been in contact with DCS in the last 6 months had 1.67 (95% CI 1.00–2.79) times the odds of PSS receipt compared to people who had not had contact with these services (Table 4). In the OPS model, people who reported last 6-month OPS access had more than twice the odds (OR 2.08 (95% CI 1.20–3.60)) of PSS access, compared to people who did not report OPS access, after adjusting for gender, age, and urbanicity (Table 5).

**Table 4:** Unadjusted and adjusted odds ratios for associations between last 6-month drug checking and prescribed safer supply receipt (*N* = 491)

	Unadjusted OR	Adjusted OR
*L6M drug checking services access*		
No	Reference	Reference
Yes	**1.76 (1.09–2.84)**	**1.67 (1.00–2.79)**
*Gender*		
Cis man	Reference	Reference
Cis woman	0.84 (0.49–1.41)	0.75 (0.43–1.30)
Transgender and gender diverse	3.01 (0.70–13.00)	4.56 (0.99–21.10)
*Age*		
< 30 years	Reference	Reference
30–39 years	**0.45 (0.22–0.93)**	0.47 (0.22–1.00)
40–49 years	0.57 (0.28–1.14)	0.60 (0.29–1.22)
≥ 50 years	**0.23 (0.10–0.49)**	**0.22 (0.10–0.51)**
Unknown	0.36 (0.07–1.73)	0.33 (0.06–1.78)
*Urbanicity*		
Small population centre	Reference	Reference
Medium population centre	0.63 (0.37–1.10)	0.75 (0.42–1.34)
Large urban population centre	**0.33 (0.18–0.63)**	**0.37 (0.19–0.73)**

Bolding reflects statistical significance, where confidence intervals do not cross 1

Adjusted models are adjusted for age, sex, urbanicity; Drug checking unadjusted model = *N* = 491; Adjusted model = *N* = 483; *N* = 8 missing on gender

**Table 5:** Unadjusted and adjusted odds ratios for associations between last 6-month overdose prevention service access and prescribed safer supply receipt (*N* = 491)

	Unadjusted OR	Adjusted OR
*L6M overdose prevention service access*		
No	Reference	Reference
Yes	1.99 (1.20–3.31)	**2.08 (1.20–3.60)**
*Gender*		
Cis man	Reference	Reference
Cis woman	0.84(0.49–1.41)	0.75(0.42–1.32)
Transgender and/or gender diverse	3.01(0.70–13.00)	4.40(0.92–20.98)
*Age*		
< 30 years	Reference	Reference
30–39 years	**0.45 (0.22–0.93)**	0.55 (0.25–1.21)
40–49 years	0.57 (0.28–1.14)	0.58 (0.27–1.26)
≥ 50 years	**0.23 (0.10–0.49)**	**0.25 (0.11–0.58)**
Unknown	0.36 (0.07–1.73)	0.19 (0.01–1.88)
*Urbanicity*		
Small population centre	Reference	Reference
Medium population centre	0.63 (0.37–1.10)	0.68 (0.37–1.25)
Large urban population centre	**0.33 (0.18–0.63)**	**0.34 (0.16–0.70)**

Bolding reflects statistical significance, where confidence intervals do not cross 1

Adjusted models are adjusted for age, sex, urbanicity; OPS unadjusted model = 443, *N* = 48 (9.8%) missing on OPS variable; *N* = 8 missing on gender, Adjusted model run on *N* = 435

After adjusting for gender, age, and urbanicity, people aged ≥ 50 years had significantly lower odds of PSS access compared to people aged < 30 years in the DCS (OR 0.22 (95% CI 0.10–0.51)) and OPS (OR 0.25 (95% CI 0.11–0.58)) models. People residing in large urban population centres had significantly lower odds of PSS access compared to people residing in small population centres in the DCS (OR 0.37 (95% CI 0.19–0.73)) and OPS (OR 0.34 (95% CI 0.16–0.70) models.

## Discussion

Overall, the proportion of HRCS respondents who received PSS is low, suggesting that PSS is not reaching all those in need in BC. This is consistent with provincial evaluations. For example, analyses from the BCCDC revealed that an estimated 6,498 people were dispensed PSS medications between March 37 2020 and February 28 2021 [5]. This is said to represent only a small proportion (< 3%) of all people who have been estimated to use drugs in the province [11]. PSS medications included in this study were prescribed opioids, stimulants, and benzodiazepines, which reflected alternatives to the substances that are commonly used by people who access the unregulated drug supply. In the present study, more than 70% of the sample reported the use of illegal opioids in the prior 3 days, and nearly 90% reported use of illegal stimulants in the prior 3 days. While interventions to reduce illegal drug toxicity deaths in BC have primarily been focused on opioid use (e.g. distribution of naloxone, increased opioid agonist treatment options) [12, 13], we found that stimulant use was even more prevalent than opioid use in this sample. Nevertheless, most of PSS prescriptions identified in this sample were for opioid PSS (68%) while a smaller proportion of people received a stimulant PSS prescription (33%). This highlights the need for increased attention to the health and service needs of people who use stimulants either alone or in combination with opioids. This attention must include considerations for expanding the available stimulant PSS options, and incorporating a range of PSS options that more closely match the preferences of people who use illegal stimulants, such as pharmaceutical cocaine [14, 15]. While this manuscript is focused on prescribed safer supply, calls have been mounting in BC for the expansion of non-prescribed models, such as compassion clubs which aim to provide a safe method for people to access substances [11, 16, 17].

Expansion of medication options is particularly important where existing studies have demonstrated that the available PSS might not match patient preferences for the potency or route of administration [18–21]. As such, a broader range of medication sub-types might be required to be prescribed, and advocacy for such expansion is ongoing [19, 22–24]. However, in the absence of currently available alternatives, PSS medications available to date have been demonstrated to have benefits in several areas of clients’ lives. For example, studies have demonstrated that access to prescribed tablet hydromorphone for people using illegal opioids reduced overdose risk, provided improvements in health and well-being, improved pain management, and led to economic improvements [25].

While not implemented under the direct framework of PSS, medications being proposed for expansion as part of PSS have a plethora of evidence to support them, from which expectations of the potential benefits of expansion of the medications can be derived. For example, there is long-standing evidence that injectable diacetylmorphine is a safe and effective treatment for opioid use disorder, and is associated with improvements in health, crime, and psychosocial outcomes [26–29]. It is important, however, to acknowledge that to date, evidence for this medication is derived from a clinical setting, where patients visit a clinic daily, which may be seen as a barrier to engagement for some people. Nevertheless, this treatment setting also comes along with shared decision-making, where patients have a choice regarding preferred medications and dose received [30], which are considerations that should be accounted for when considering the potential of PSS to meet the needs of those it is meant to serve.

While access to preferred versions of PSS is needed to support separation from the unregulated drug supply, the rapid expansion of a wider range of medications to meet preferences via PSS has not yet occurred. In the absence of broadly available PSS options however, there may still be a role for currently available options. For example, in a study of patient preferences in injectable OAT, more than 80% of patients preferred diacetylmorphine over hydromorphone, but more than 80% also said they would still take hydromorphone if diacetylmorphine was not available [17, 31]. The lack of action on expansion of PSS options should not deter the broader implementation of currently available options for those who want them. This expansion can occur recognizing the limitations of these options, without sacrificing continued advocacy for a wider range of options, which could include injectable or inhalable versions of opioids to match growing rates of smoking among people who use drugs in BC [32].

Furthermore, in the absence of expansion of new medications, there are aspects of implementation of currently available medications that could be shifted to better meet needs. For example, a qualitative study of patients receiving dextroamphetamine (prescription psychostimulant) found that patients wanted to receive higher doses, with faster titration, and have access to take-home doses [33]. There is growing evidence to support the safety and effectiveness of prescribed psychostimulants when provided in robust doses (> 60 mg or more per day) [34]. Emerging evidence suggests these doses are often not reached in practice, and some patients will need higher doses than listed in PSS guidance to achieve an effect [22]. Prescription psychostimulants may not meet the needs of people who do not want to achieve abstinence, and who are seeking effects similar to those gained from the use of cocaine or methamphetamine, highlighting the urgency and need for expansion of alternative PSS options. Such alternatives could include medications like Desoxyn (prescribed methamphetamine) and drugs available through non-prescribed safer supply models.

We found that more than 60% of the sample reported the use of both opioids and stimulants, reflecting high rates of co-use of these substances, which have been reported more widely in population-level samples in BC, Canada, and North America [35–39]. People are known to have a range of motivations for co-use [40] and to practice strategies to protect from harm [41], nevertheless the risk of harm persists given the rising toxicity of the unregulated drug supply in BC [42]. Despite this co-use being common, only 30% of people who received an opioid PSS prescription in this study also received a stimulant PSS prescription. This highlights the need for increased resources for people who engage in polysubstance use and more efforts to determine how concurrent use of stimulants and opioids can be addressed through a combination of PSS and adjacent harm reduction and substance use treatment services that meet client goals. Harm reduction services and broader health system contact is particularly important for this population, given studies have identified elevated risk of a number chronic disease diagnoses for people with concurrent opioid and stimulant use disorders [43], and co-use of opioids and stimulants has been associated with increased risk of infectious disease [44].

In this study, we identified that DCS and OPS were highly correlated. Prior studies have shown that drug checking is associated with OPS/SCS use (Tobias), which is intuitive given all OPS have at minimum, test strips. Some sites also have part-time spectrometers on site for drug checking. DCS are not offered exclusively at SCS/OPS sites, however most spectrometers are offered at these sites. In sites where DCS are offered in the context of an OPS/SCS, the hope is that participants will check their drugs prior to consuming them, but services are available regardless of subsequent OPS use. In sites where there is no consumption space on site, DCS are considered drop-in, with supply pick-up always available. Some sites in rural communities also have designated mail-in services. There are many service models for in-person drug checking which are mostly flexible.

We also identified that contact with DCS or OPS in the prior 6 months was associated with access to PSS. Prior studies have similarly identified that people who are more regularly engaged in services are more likely to access PSS. For example, a study of clients receiving opioid PSS identified that those who were receiving mental health medications, and those who were engaged in OAT had higher odds of PSS adherence [45]. Similarly, OAT has been identified as a predictor of PSS awareness among people who use unregulated drugs in BC [46]. Finding may reflect the important role people working at OPS and DCS sites play in connecting clients to ancillary harm reduction and treatment services such as PSS. The role of people with lived and living experience of substance use in overdose response in BC must be acknowledged, where many DCS and OPS services rely on the expertise and leadership of people working in what are often termed “Peer worker” roles [47–50]. Studies have demonstrated that peer workers rely on their informal roles and social networks to develop a sense of trust and safety that cannot be met by non-peer staff [51, 52]. Scaling up of programmes that rely on peer workers may support increased harm reduction service connection and may support referral to and engagement with PSS.

Findings of this study suggest that additional outreach strategies and service models are needed to reach people who are not already connected to services and to improve the accessibility of harm reduction services (i.e. increased service hours, and reduced wait lists). In this study we found that rates of access to PSS were higher in Northern Health, as compared to other Health Authorities. Regional differences identified in this study are not representative of provincial trends, however they do highlight the efforts in specific communities to reach and engage people at harm reduction sites with access to PSS. For example, the high rates of PSS access in Northern Health are understood to be attributable to a peer-led model in this health region, in which a person with lived experience of substance use supports clients to connect to a PSS prescriber and facilitates medication access through a low-barrier medication delivery programme [53].

Interventions such as this peer-led programme can serve as a model that can be implemented in other settings across the province to adopt responses to better meet and reach the needs of people at risk of overdose. Such an approach would reflect responsiveness to the needs of the community during dual public health crises. A recent environmental scan of PSS programmes in BC has reflected that such flexibility is possible and has identified that changes to service provision have been significant since PSS was first introduced in March 2020 [54]. For example, the scan identified several examples of changes to staffing, physical spaces, programme operations, and client protocols in PSS programmes, with the primary goal of reducing COVID-19 infection and overdose risk. These high rates of adaptation demonstrate that health system changes are possible to promote client connections to PSS in BC. Such changes could be implemented to reach clients who remain the least engaged in care, including people accessing harm reduction services, where PSS rates remain low, and for people who are disconnected from both harm reduction and treatment services in in the province.

## Limitations

Findings should be interpreted relative to the context from where the data were derived. The HRCS is a convenience sample and therefore is not representative of the distribution of PSS across the province. Because the HRCS samples people who are attending harm reduction sites, these results reflect the responses of people who are already receiving some health care services. Caution should be taken in extending conclusions to populations with less connection to the health care system.

Participants were eligible for the analysis if they reported any illegal opioid use, illegal stimulant use, or any benzodiazepine use in the prior 3 days. This inclusion criterion was applied to ensure the sample included only those who were eligible to receive an opioid, stimulant, or benzodiazepine PSS prescription at the time of survey completion. There were seven participants who reported PSS receipt who did not report use of the substances listed above in the last 3 days and thus were not included in the analysis. We cannot disentangle whether PSS had separated these participants from the unregulated drug supply.

It is possible that access to PSS could reduce the frequency of contact with harm reduction services, and therefore people receiving PSS might not be well engaged in the harm reduction sites sampled in this study. It is therefore possible that we are underrepresenting the proportion of people receiving PSS who would typically be in contact with harm reduction services (had they not received PSS). Nevertheless, this survey is a convenience sample of people accessing harm reduction services in BC and is not intended to be representative of all people who use drugs in BC, nor of all people accessing PSS in BC. Furthermore, the hypothesis that access to PSS might limit the need for connection to harm reduction services is not yet well-supported by data. Given the known gaps in available PSS to date (i.e. with respect to matching preferences for medication type, route of use, and dose), we cannot expect that access to PSS is synonymous with cessation of unregulated substance use, or cessation of contact with harm reduction services.

This is a cross-sectional survey and therefore we cannot draw any conclusions about temporal relationships between the receipt of PSS and illegal substance use. Because of the way the questions were posed, we don’t know when participants received PSS medications (i.e. past, currently, etc.), nor for how long they received PSS (i.e. one time prescription, long-term prescription). Other important data related to the dispensation of PSS medication (e.g. daily dispense) is not available in this study and should be further explored with administrative data.

Furthermore, data on history of incarceration and recent contact with the criminal legal system were not collected in this study and could be prioritized in future studies, given the known associations between incarceration and elevated overdose risk in BC [55, 56] and barriers to community health, harm reduction, and treatment services facing this population [57–59].

We found a high proportion of participants reporting the use of both opioids and stimulants. While other patterns of co-use existed (e.g. concurrent use of opioids and benzodiazepines), the reported co-use of these substances was less prevalent and was not a focus of this study. Future research on PSS should incorporate a focus on patterns of co-use of these substances, particularly given the increasing contamination of the opioid supply with unregulated benzodiazepines and known increased risk of mortality with co-use of two substances. In the context of a rapidly changing unregulated drug supply, future research may also draw on qualitative and ethnographic research methods to better understand the changing patterns of use and PSS preferences of people who use drugs in BC in real time.

## Conclusion

Overall, the proportion of respondents who received PSS was low, suggesting that this intervention is not reaching all those in need. Harm reduction services may serve as a point of contact for referral to PSS. Additional outreach strategies and service models are needed to improve the accessibility of harm reduction services and of prescribed safer supply in British Columbia.

## Data Availability

The datasets generated during and/or analysed during the current study are not publicly available but may be available from the corresponding author on reasonable request.

## Abbreviations

BC: British Columbia
CI: Confidence interval
DCS: Drug checking service
HRCS: Harm Reduction Client Survey
iOAT: Injectable opioid agonist treatment
OAT: Opioid agonist treatment
OPS: Overdose prevention service
OUD: Opioid use disorder
OR: Odds ratio
PSS: Prescribed safer supply
REB: Research ethics board
SCS: Supervised consumption sites

## References

[1] Palis H, Bélair MA, Hu K, Tu A, Buxton J, Slaunwhite A (2021). Overdose deaths and the COVID-19 pandemic in British Columbia, Canada. Drug Alcohol Rev.

[2] BC Coroners Service. Illicit drug toxicity deaths in BC. 2022.

[3] British Columbia Ministry of Health, British Columbia Centre on Substance Use. Risk Mitigation in the context of dual public health Emergencies. https://www.bccsu.ca/wp-content/uploads/2020/04/Risk-Mitigation-in-the-Context-of-Dual-Public-Health-Emergencies-v1.5.pdf. 2020.

[4] Nosyk B, Slaunwhite A, Urbanoski K, Hongdilokkul N, Palis H, Lock K (2021). Evaluation of risk mitigation measures for people with substance use disorders to address the dual public health crises of COVID-19 and overdose in british Columbia: a mixed-methods study protocol. BMC Health Serv.

[5] Ministry of Health, Ministry of mental health and addictions. Access to prescribed safer supply in british columbia: Policy direction. 2021.

[6] BC Centre for Disease Control. Unregulated drug poisoning emergency dashboard 2023 Available from: http://www.bccdc.ca/health-professionals/data-reports/substance-use-harm-reduction-dashboard

[7] Tupper KW, McCrae K, Garber I, Lysyshyn M, Wood E (2018). Initial results of a drug checking pilot program to detect fentanyl adulteration in a Canadian setting. Drug Alcohol Depend.

[8] Potier C, Laprevote V, Dubois-Arber F, Cottencin O, Rolland B (2014). Supervised injection services: What has been demonstrated? A systematic literature review. Drug Alcohol Depend.

[9] Semaan S, Fleming P, Worrell C, Stolp H, Baack B, Miller M (2011). Potential role of safer injection facilities in reducing HIV and hepatitis C infections and overdose mortality in the United States. Drug Alcohol Depend.

[10] BC Centre for Disease Control. BC Harm reduction client survey 2021 [Available from: http://www.bccdc.ca/resource-gallery/Documents/Statistics%20and%20Research/Statistics%20and%20Reports/Overdose/2021%20-%20BC_Overall_HR%20Survey%20%28Apr%2020%29.pdf.

[11] BC Coroners Service. BC coroners service death review panel: an urgent response to a continuing crisis. 2023.

[12] Moe J, Godwin J, Purssell R, O'Sullivan F, Hau JP, Purssell E (2020). Naloxone dosing in the era of ultra-potent opioid overdoses: a systematic review. CJEM.

[13] Pearce LA, Min JE, Piske M, Zhou H, Homayra F, Slaunwhite A (2020). Opioid agonist treatment and risk of mortality during opioid overdose public health emergency: population based retrospective cohort study. BMJ.

[14] Buxton J, Xavier J. Perspectives on safer supply: insights from people who use substances in British Columbia. 2023.

[15] Grzybowski A (2007). The history of cocaine in medicine and its importance to the discovery of the different forms of anaesthesia. Klin Oczna.

[16] BC Centre on Substance Use. Heroin compassion clubs. 2022.

[17] Podcast C. 2023. Podcast. Available from: https://www.crackdownpod.com/episodes/zir7c16nb6j8xkm7tlbi3wd3s5j3d4

[18] Ferguson M, Parmar A, Papamihali K, Weng A, Lock K, Buxton JA (2022). Investigating opioid preference to inform safe supply services: a cross sectional study. Int J Drug Policy.

[19] Bardwell G (2022). More than a pipe dream? The need to adapt safer opioid supply programs for people who smoke drugs. J Stud Alcohol Drugs.

[20] Kamal A, Ferguson M, Xavier JC, Liu L, Graham B, Lock K (2023). Smoking identified as preferred mode of opioid safe supply use; investigating correlates of smoking preference through a 2021 cross-sectional study in British Columbia. Subst Abuse Treat Prev Policy.

[21] Ferguson M, Sedgemore KO, Scow M, Choisil P, Haywood B, Xavier J (2023). Preferred stimulant safer supply and associations with methamphetamine preference among people who use stimulants in British Columbia: findings from a 2021 cross-sectional survey. Int J Drug Policy.

[22] Brothers TD, Leaman M, Bonn M, Lower D, Atkinson J, Fraser J, Gillis A, Gniewek LH, Hayman H, Jorna P, Martell D, O'Donnell T, Bowerman H, Genge L (2022). Evaluation of an emergency safe supply drugs and managed alcohol program in COVID-19 isolation hotel shelters for people experiencing homelessness. Drug Alcohol Depend.

[23] Bonn M, Palayew A, Bartlett S, Brothers TD, Touesnard N, Tyndall M (2021). "The times they are a-changin": addressing common misconceptions about the role of safe supply in north America’s overdose crisis. J Stud Alcohol Drugs.

[24] Bonn M, Touesnard N, Puliese M, Cheng B, Comeau E, Bodkin C, Brothers T, Wildeman S. Securing safe supply during COVID-19 and beyond: scoping review and knowledge mobilization. 2021.

[25] Ivsins A, Boyd J, Mayer S, Collins A, Sutherland C, Kerr T (2021). "It’s helped me a lot, just like to stay alive": a qualitative analysis of outcomes of a novel hydromorphone tablet distribution program in Vancouver, Canada. J Urban Health.

[26] Oviedo-Joekes E, Brissette S, Marsh DC, Lauzon P, Guh D, Anis A (2009). Diacetylmorphine versus methadone for the treatment of opioid addiction. N Engl J Med.

[27] Oviedo-Joekes E, Guh D, Brissette S, Marchand K, MacDonald S, Lock K (2016). Hydromorphone compared with diacetylmorphine for long-term opioid dependence: a randomized clinical trial. JAMA Psychiat.

[28] Palis H, Marchand K, Guh D, Brissette S, Lock K, MacDonald S (2017). Men's and women's response to treatment and perceptions of outcomes in a randomized controlled trial of injectable opioid assisted treatment for severe opioid use disorder. Subst Abuse Treat Prev Policy.

[29] Strang J, Groshkova T, Uchtenhagen A, van den Brink W, Haasen C, Schechter MT (2015). Heroin on trial: systematic review and meta-analysis of randomised trials of diamorphine-prescribing as treatment for refractory heroin addictiondagger. Br J Psychiatry.

[30] Marchand K, Foreman J, MacDonald S, Harrison S, Schechter MT, Oviedo-Joekes E (2020). Building healthcare provider relationships for patient-centered care: a qualitative study of the experiences of people receiving injectable opioid agonist treatment. Subst Abuse Treat Prev Policy.

[31] Oviedo-Joekes E, Marchand K, Palis H, Guh D, Brissette S, Lock K (2016). Predictors of treatment allocation guesses in a randomized controlled trial testing double-blind injectable hydromorphone and diacetylmorphine for severe opioid use disorder. Addict Res Theory.

[32] Parent S, Papamihali K, Graham B, Buxton JA (2021). Examining prevalence and correlates of smoking opioids in British Columbia: opioids are more often smoked than injected. Subst Abuse Treat Prev Policy.

[33] Palis H, Marchand K, Peachy G, Westfall J, Lock K, Macdonald S, Harrison S, Jun J, Bojanczyk-Shibata A, Marsh DC, Schechter MT, Oviedo-Joekes E (2021). Exploring the effectiveness of dextroamphetamine for the treatment of stimulant use disorder: a qualitative study with injectable opioid agonist treatment patients. Subst Abuse Treat Prev. Policy.

[34] Tardelli VS, Bisaga A, Arcadepani FB, Gerra G, Levin FR, Fidalgo TM (2020). Prescription psychostimulants for the treatment of stimulant use disorder: a systematic review and meta-analysis. Psychopharmacology.

[35] Government of Canada. Opioid and stimulant related harms in Canada 2022. Available from: https://health-infobase.canada.ca/substance-related-harms/opioids-stimulants/

[36] Palis H, Xavier C, Dobrer S, Desai R, Sedgemore KO, Scow M (2022). Concurrent use of opioids and stimulants and risk of fatal overdose: A cohort study. BMC Public Health.

[37] Lukac CD, Steinberg A, Papamihali K, Mehta A, Lock K, Buxton JA (2022). Correlates of concurrent use of stimulants and opioids among people who access harm reduction services in British Columbia, Canada: findings from the 2019 harm reduction client survey. Int J Drug Policy.

[38] Steingberg A, Mehta A, Lukac C, Buxton J (2020). Exploring motivations for concurrent use of uppers and downers among people who access harm reduction services in BC: a thematic analysis based on findings from the 2019 HRCS. BMJ Open.

[39] Ellis MS, Kasper ZA, Cicero TJ (2018). Twin epidemics: the surging rise of methamphetamine use in chronic opioid users. Drug Alcohol Depend.

[40] Steinberg A, Mehta A, Papamihali K, Lukac CD, Young S, Graham B (2022). Motivations for concurrent use of uppers and downers among people who access harm reduction services in British Columbia, Canada: findings from the 2019 harm reduction client survey. BMJ Open.

[41] Corser J, Palis H, Fleury M, Lamb J, Lock K, McDougall J (2022). Identifying behaviours for survival and wellness among people who use methamphetamine with opioids in British Columbia: a qualitative study. Harm Reduct J.

[42] Palis H, Tu A, Scow M, Young P, Wood S, Hu K, Slaunwhite AK et al. Increasing toxicity of the illicit drug supply during COVID-19: the need for an accessible and acceptable safe supply. UBC Med J. 2022;13.

[43] Palis H, Gan W, Xavier C, Desai R, Scow M, Sedgemore KO (2022). Association of opioid and stimulant use disorder diagnoses with fatal and nonfatal overdose among people with a history of incarceration. JAMA Netw Open.

[44] Serota DP, Bartholomew TS, Tookes HE (2021). Evaluating differences in opioid and stimulant use-associated infectious disease hospitalizations in Florida, 2016–2017. Clin Infect Dis.

[45] Selfridge M, Card K, Kandler T, Flanagan E, Lerhe E, Heaslip A (2022). Factors associated with 60-day adherence to “safer supply” opioids prescribed under British Columbia’s interim clinical guidance for health care providers to support people who use drugs during COVID-19 and the ongoing overdose emergency. Int J Drug Policy.

[46] Moshkforoush M, DeBeck K, Brar R, Fairbairn N, Cui Z, Milloy MJ (2022). Low awareness of risk mitigation prescribing in response to dual crises of COVID-19 and overdose deaths among people who use unregulated drugs in Vancouver, Canada. Harm Reduct J.

[47] Mamdani Z, Feldman-Kiss D, McKenzie S, Knott M, Cameron F, Voyer R (2022). Core competencies of peer workers who use pulse oximeters to supplement their overdose response in British Columbia. PLoS ONE.

[48] Kennedy MC, Boyd J, Mayer S, Collins A, Kerr T, McNeil R (2019). Peer worker involvement in low-threshold supervised consumption facilities in the context of an overdose epidemic in Vancouver, Canada. Soc Sci Med.

[49] Greer A, Buxton JA, Pauly B, Bungay V (2021). Organizational support for frontline harm reduction and systems navigation work among workers with living and lived experience: qualitative findings from British Columbia, Canada. Harm Reduct J.

[50] Pauly BB, Mamdani Z, Mesley L, McKenzie S, Cameron F, Edwards D (2021). “It’s an emotional roller coaster… but sometimes it’s fucking awesome”: meaning and motivation of work for peers in overdose response environments in British Columbia. Int J Drug Policy.

[51] Bardwell G, Kerr T, Boyd J, McNeil R (2018). Characterizing peer roles in an overdose crisis: preferences for peer workers in overdose response programs in emergency shelters. Drug Alcohol Depend.

[52] Scow M, McDougall J, Slaunwhite A, Palis H (2023). Peer-led safer supply and opioid agonist treatment medication distribution: a case study from rural British Columbia. Harm Reduct J.

[53] Day T, de Kock H, Matieschyn Z, McDougall J. Rural webinar- risk mitigation and OUD practice update BC centre on substance use practice update. 2022.

[54] McCrae K, Glegg S, Goyer M, Le Foll B, Brar R, Sutherland C (2022). The changing landscape of pharmaceutical alternatives to the unregulated drug supply during COVID-19. Harm Reduct J.

[55] Keen C, Kinner SA, Young JT, Snow K, Zhao B, Gan W (2021). Periods of altered risk for non-fatal drug overdose: a self-controlled case series. Lancet Public Health.

[56] Gan WQ, Kinner SA, Nicholls TL, Xavier C, Urbanoski K, Greiner L (2020). Risk of overdose-related death for people with a history of incarceration. Addiction.

[57] Palis H, Hu K, Rioux W, Korchinski M, Young P, Greiner L (2022). Association of mental health services access and reincarceration among adults released from prison in British Columbia, Canada. JAMA Netw Open.

[58] Palis H, Zhao B, Young P, Korchinski M, Greiner L, Nicholls T (2022). Stimulant use disorder diagnosis and opioid agonist treatment dispensation following release from prison: a cohort study. Subst Abuse Treat Prev Policy.

[59] Kouyoumdjian FG, Cheng SY, Fung K, Orkin AM, McIsaac KE, Kendall C (2018). The health care utilization of people in prison and after prison release: a population-based cohort study in Ontario, Canada. PLoS ONE.

